# O10.6. OLANZAPINE IMPAIRS CENTRAL INSULIN ACTION: EFFECTS ON BODY FUEL PREFERENCE IN RATS

**DOI:** 10.1093/schbul/sby015.258

**Published:** 2018-04-01

**Authors:** Laura Castellani, Jennifer Wilkin, Zohra Ahsan, Celine Teo, Chantel Kowalchuk, Adria Giacca, Gary Remington, Margaret Hahn

**Affiliations:** 1 Centre for Addiction and Mental Health; 2 University of Toronto

## Abstract

**Background:**

Antipsychotics (APs) remain the cornerstone of treatment in schizophrenia, with increasing use on- and off- label. Olanzapine (OLZ) is a highly prescribed, high metabolic liability AP. Olanzapine has also been found to shift major fuel preference from carbohydrates to fats (while impairing fat breakdown and decreasing availability of fat as a substrate). The shift toward fat utilization is demonstrated by decreases in respiratory exchange ratio (RER). Notably, APs may alter signaling pathways in insulin-sensitive brain areas, particularly in the hypothalamus, that play a role glucose regulation and energy expenditure.

**Methods:**

We investigated the effects of intracerebroventricular (ICV) insulin administration on OLZ-induced disruptions in energy homeostasis. Male Sprague Dawley rats were assigned to 4 treatment groups (ICV-peripheral): Vehicle (VEH)-VEH (n = 5), Insulin (INS)-VEH (n = 7), INS-OLZ (n = 6), VEH-OLZ (n = 5). Following acclimatization to the metabolic cages, rats received injections of INS (10mU) or VEH into the 3rd ventricle, and OLA (3mg/kg) or VEH subcutaneously at the beginning of the light (7AM, t=0) and dark (7PM, t=12h) cycle. Dose of OLZ was chosen based on clinically relevant >65% dopamine (D2) brain occupancy. Dose of ICV insulin was chosen based on established decreases in food intake. Indirect calorimetry was used to calculate RER, and heat production. Cumulative food intake was measured at 12-hour intervals (t=12h and 24 h).

**Results:**

Treatment with OLZ reproduced the previously established downward shift in RER during the dark phase (p=0.016), which occurred independently of changes in food intake or heat production. Central insulin also decreased RER (p=0.013), an unexpected finding as insulin has been associated with increased carbohydrate oxidation. This may have been secondary to decreased food intake associated with central INS (p=0.011), leading to a shift towards fat-oxidation characteristic of fasting. Co-administration of OLZ with central INS (INS-OLZ) abolished the treatment effect seen in the INS-VEH group relative to VEH-OLZ, shifting the RER profile to become similar to the VEH-OLZ group. Similarly, when OLZ was co-administered with ICV-INS, the effect of central INS to reduce food-intake was lost. An interaction effect (p=0.007) was observed between central insulin and subcutaneous OLZ treatments on RER, supporting the notion that these compounds may work through independent mechanisms to influence fuel preference.

**Discussion:**

Taken together, these findings suggest that: 1) central insulin stimulation alters metabolism but is unable to modulate OLZ-specific associated changes in RER; 2) this is likely occurring due to rapid induction of central insulin resistance by OLZ. Our data thus warrants further investigation into the effects of APs on central insulin sensing, and mitigation strategies (i.e. use of central insulin sensitizers) with the goal of mitigating the metabolic burden of these compounds.

